# Octahedral Tilting in Homologous Perovskite Series CaMoO3-SrMoO3-BaMoO3 Probed by Temperature-Dependent EXAFS Spectroscopy

**DOI:** 10.3390/ma15217619

**Published:** 2022-10-30

**Authors:** Georgijs Bakradze, Alexei Kuzmin

**Affiliations:** Institute of Solid State Physics, University of Latvia, 8 Kengaraga Street, LV-1063 Riga, Latvia

**Keywords:** CaMoO_3_, SrMoO_3_, BaMoO_3_, perovskite, octahedral tilting, EXAFS spectroscopy, reverse Monte Carlo

## Abstract

Local distortions in perovskites can be induced by cation displacements and/or by the tilting and rotating of cation–anion octahedra. Both phenomena have been subject to intense investigations over many years. However, there are still controversies in the results obtained from experimental techniques that are sensitive to long-range order (X-ray, neutron, or electron diffraction) and those sensitive to short-range order (X-ray absorption spectroscopy). In this study, we probed the details of the local environment in *A*MoO_3_ perovskites (*A* = Ca, Sr, Ba) using extended X-ray absorption fine structure (EXAFS) in a wide temperature range (10–300 K). An advanced analysis of the EXAFS spectra within the multiple-scattering formalism using the reverse Monte Carlo method enhanced by an evolutionary algorithm allowed us (i) to extract detailed information on metal–oxygen and metal–metal radial distribution functions, and metal–oxygen–metal and oxygen–metal–oxygen bond angle distribution functions, and (ii) to perform polyhedral analysis. The obtained results demonstrate the strong sensitivity of the EXAFS spectra to the tilting of [MoO_6_] octahedra induced by the differences in the sizes of alkaline earth metal cations (Ca^2+^, Sr^2+^, and Ba^2+^).

## 1. Introduction

Over the last few years, various interesting physical properties have been explored in 4D transition-metal perovskites. In particular, molybdenum-based perovskites with a general composition *A*MoO_3_ (*A* = Ca, Sr, Ba) exhibit low work function (e.g., only 1.1 eV for BaMoO_3_ [1]) and the lowest specific resistivity among oxides (e.g., 5.1 μΩ cm for SrMoO_3_ at room temperature [2]), performing well as oxidants, reducers, etc. To date, the perovskite molybdates—or trioxomolybdates of the alkaline earth metals—have been synthesized as powders [3], thin films [4,5], and single crystals [2], thus, opening new paths for producing nontoxic, earth-abundant, Mo-based, correlated metal perovskite-type oxides.

*A*MoO_3_ (*A* = Ca, Sr, Ba) perovskites contain Mo^4+^(4d^2^) ions with two electrons in the partially filled $t_{2g}$ band and, therefore, exhibit metallic conductivity and Pauli paramagnetic properties similar to those of ReO_3_ [6,7]. At the same time, their crystallographic structure depends on the size of an $A^{2+}$ cation, which increases from Ca^2+^ to Ba^2+^ [8]. The effective ionic radii of twelvefold coordinated Ca^2+^, Sr^2+^, and Ba^2+^ ions are 1.34 Å, 1.44 Å, and 1.61 Å, respectively [8].

**Figure 1:**
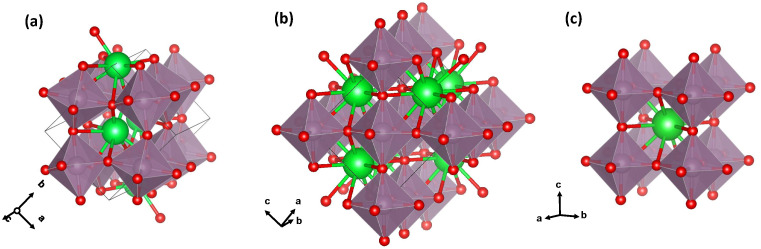
Crystal structures of (**a**) CaMoO_3_, (**b**) SrMoO_3_ (at temperatures below 125 K), and (**c**) BaMoO_3_. The conventional crystallographic axes have been rotated to emphasize the similarity of the structures, which consist of a network of corner-shared [MoO_6_] octahedra.

According to diffraction studies at room temperature [9,10], SrMoO_3_ and BaMoO_3_ crystallize in the cubic perovskite structure with the space group $Pm\;\bar{\;3\;}\;m$ (Z=1, no. 221), which is composed of a 3D network of regular corner-linked [MoO_6_] octahedra (see Figure 1). Mo ions reside at the centre of octahedra, whereas *A* ions (Sr or Ba) are located in a body-centred cube formed by eight corner-linked octahedra. The octahedral axes formed by Mo–O bonds coincide with the cubic lattice axes, and the only variable structural parameter is the lattice parameter.

SrMoO_3_ undergoes two phase transitions, which are characterized by a gradual increase in the rotation angle with decreasing temperatures [11]: at temperatures between 125 K and 266 K, the out-of-phase [MoO_6_] tilting results in the tetragonal I4/mcm (Z=4, no. 140) phase and at temperatures below 125 K in an orthorhombic Imma (Z=4, no. 74) phase [11]. We note here that neither X-ray nor neutron diffraction had given any hints on the possible deviation of the local symmetry at the Mo sites from $O_{h}$ in SrMoO_3_ and BaMoO_3_.

In CaMoO_3_, the lattice symmetry is lowered from cubic to orthorhombic Pbnm (Z=4, no. 62) by a cooperative tilting of [MoO_6_] octahedra [3,12] (see Figure 1). This distortion is driven by the mismatch between the size of the cubo-octahedral cavity in the corner-sharing octahedral network and a relatively small ionic radius of the Ca^2+^ ions [8]. Octahedral tilting opens up the possibility of a slight off-centre displacement of Ca^2+^ ions in the ab-plane to reduce the tension in Ca–O bonds so that the group of 12 oxygen atoms located in the first coordination shell of calcium in the cubic phase splits into two groups of the eight nearest (2.37–2.71 Å) and four distant (3.13–3.39 Å) oxygen atoms [12]. The tilting of octahedra maximizes the *A*–O covalent bonding and minimizes the repulsive *A*–O overlap in the system [13,14,15].

In perovskites, the doping or softening of vibrational modes can occasionally lead to lattice distortion from the cubic structure. Three types of distortions can be identified: (i) distortions of octahedral units, (ii) cation displacements within the octahedra, and (iii) octahedral tilting distortions [16]. The deviation from cubic geometry in perovskites correlates with their physical properties so understanding and controlling them requires paying careful attention to structural distortions [17,18,19]. Although diffraction methods provide information on the averaged long-range order, X-ray absorption spectroscopy (XAS) is sensitive to the short-range surroundings of the respective element and, therefore, is well suited to probe local deviations from the average symmetry [20,21,22,23,24].

In this study, we focus on the local atomic structure in three *A*MoO_3_ perovskites with different types of structural distortions. Tilted [MoO_6_] octahedra are present in CaMoO_3_, BaMoO_3_ has a regular perovskite structure with only thermal disorder effects, and an intermediate situation occurs in SrMoO_3_. The reverse Monte Carlo method with the evolutionary algorithm (RMC/EA) [25] was used to reconstruct the structure of the perovskites at three different temperatures (10, 150, and 300 K) using the Mo K-edge (and the Sr K-edge for SrMoO_3_) extended X-ray absorption fine structure (EXAFS) spectra. As a result, we demonstrated the ability of EXAFS spectroscopy to provide useful information on the octahedral tilting distortions in perovskites in terms of the atomic radial and angle distribution functions.

## 2. Materials and Methods

Polycrystalline *A*MoO_3_ samples were produced by a reduction of *A*MoO_4_ in an Ar + 3% H_2_ (flow 12.5 Nl/h) atmosphere at high temperatures (900 ${}^{{}^{\circ}}$C, 1050 ${}^{{}^{\circ}}$C, and 1150 ${}^{{}^{\circ}}$C for *A* = Ca, Sr, and Ba, respectively) for 10 h. After synthesis, the *A*MoO_3_ powders were checked for phase purity by XRD and immediately sealed in quartz tubes under vacuum to avoid oxidation. The *A*MoO_4_ precursor powders were synthesized by mixing stoichiometric amounts of aqueous solutions of (NH_4_)_6_Mo_7_O_24_·4H_2_O and CaCl_2_, Sr(NO_3_)_2_·6H_2_O or Ba(NO_3_)_2_·6H_2_O salts at room temperature (20 ${}^{{}^{\circ}}$C) and pH = 10, followed by an annealing of the filtered and washed precipitates at 650 ${}^{{}^{\circ}}$C in air.

X-ray absorption spectroscopy (XAS) experiments were performed at the PETRA III P65 undulator beamline [26]. The X-ray absorption spectra at the Mo K-edge (20,000 eV)—for SrMoO_3_ also at the Sr K-edge (8979 eV)—were collected in transmission mode using two ionization chambers filled with argon and krypton gases. The storage ring operated at an energy of 6.08 GeV and a current of 120 mA in top-up 480 bunch mode. The harmonic reduction was achieved by uncoated (Sr K-edge) and Rh-coated (Mo K-edge) silicon plane mirrors. Fixed-exit double crystal monochromators Si(111) and Si(311) were used. The powder samples were milled in an agate mortar with cellulose and, next, were pressed into pellets. The sample weight was chosen to result in an absorption edge jump close to 1.0.

The measurements were performed at three temperatures (10, 150, and 300 K) using the Janis Research Company, LLC liquid helium flow cryostat, allowing the sample temperature to stabilize at each temperature for 20 min.

**Figure 2:**
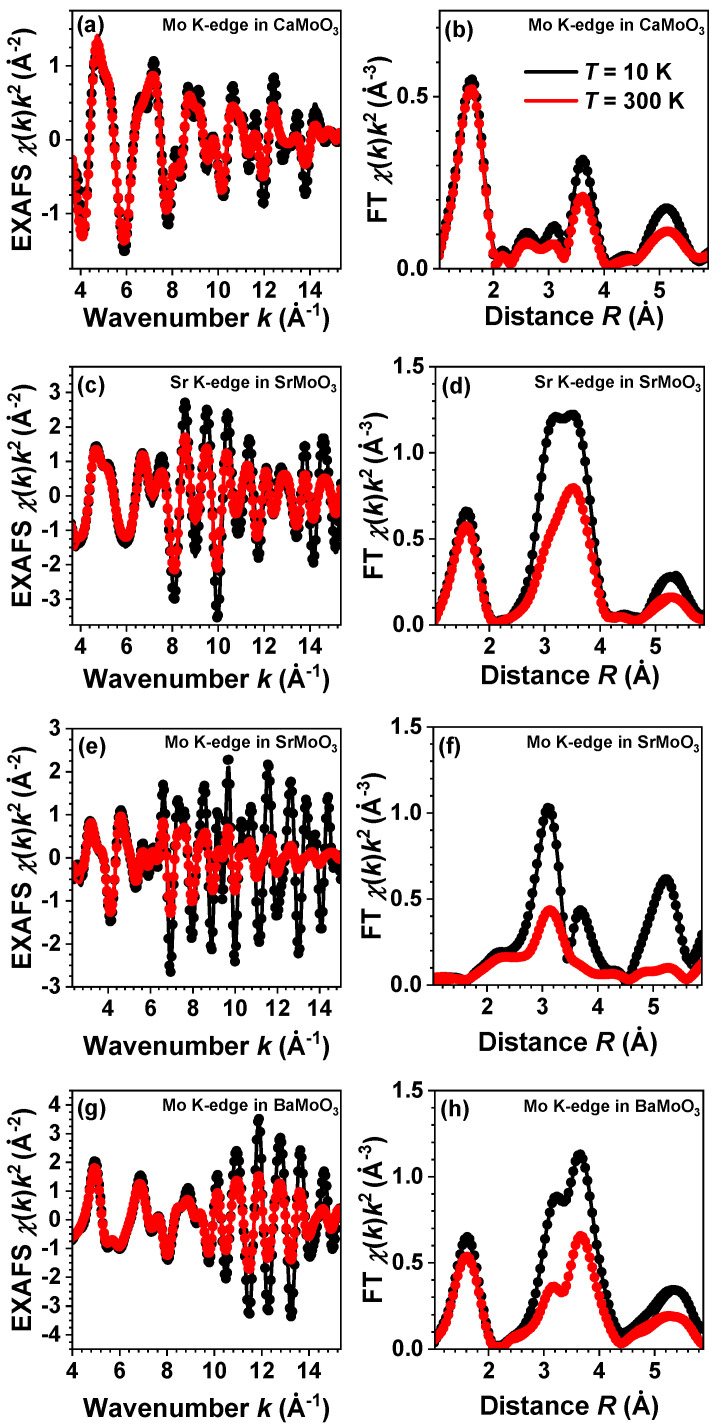
Experimental (dots) and simulated (solid lines) Mo K-edge extended X-ray absorption fine structure (EXAFS) spectra $\chi \left(k\right)k^{2}$ (left panels) and their Fourier transforms (FTs) (right panels) at 10 K (black) and 300 K (red) in CaMoO_3_ (**a**,**b**), SrMoO_3_ (**e**,**f**), and BaMoO_3_ (**g**,**h**). The data for Sr K-edge in SrMoO_3_ are shown in (**c**,**d**). Only the moduli of the FTs are shown. See text for details.

The EXAFS spectra $\chi \left(k\right)k^{2}$ were extracted using the conventional procedure as the function of the photoelectron wavenumber $k=\sqrt{\left(2m_{e}/\hbar^{2}\right)\left(E-E_{0}\right)}$, where *E* is the X-ray photon energy; $E_{0}$ is the threshold energy, i.e., the energy of a free electron with zero momentum; $m_{e}$ is the electron mass; and *ℏ* is the reduced Planck’s constant [27]. A comparison of several EXAFS measurements performed for each sample indicated that the statistical noise in the EXAFS spectra was much lower than the systematic uncertainty of data processing. The EXAFS spectra and their Fourier transforms (FTs) at three temperatures are shown in Figure 2. The FTs were calculated in the *k*-space range of 1.3–12.0 Å${}^{-1}$ for the Sr K-edge and 2.4–15.0 Å${}^{-1}$ for the Mo K-edge. The FTs were not corrected for the backscattering phase shift of atoms; therefore, the positions of all peaks in the FTs were shifted to smaller distances relative to their crystallographic values.

The EXAFS spectra were analyzed using the RMC/EA method implemented in the EvAX code ([25]). The method was successfully employed in the past to study compounds with a perovskite-type structure such as ReO_3_ [28], SrTiO_3_ [29], Cu_3_N [30], CaFeO_3_, and SrFeO_3_ [31].

The initial structural model in the RMC/EA simulations was a supercell with a 5a×5b×5c size (*a*, *b*, and *c* are the lattice parameters determined by diffraction). It was constructed based on the crystallographic structures of CaMoO_3_ [3,12], SrMoO_3_ [11], and BaMoO_3_ [9,10].

The atomic coordinates were changed randomly during the iterative process until the difference between the Morlet wavelet transforms [32] of the experimental and configuration-averaged (CA) EXAFS spectra was minimized. This allows one to achieve a good agreement simultaneously in both the *k*- and *R*- spaces. Note that one structural model was simultaneously fitted to both the Sr and Mo K-edges for SrMoO_3_. The comparison of the experimental and CA EXAFS spectra was performed in the *k*-space range of 3.0–10.0 Å${}^{-1}$ for the Sr K-edge in SrMoO_3_ and 3.0–16.0 Å${}^{-1}$ for the Mo K-edge in all compounds and in the *R*-space range of 1.0–5.5 Åfor the Sr K-edge and 1.0–6.5 Åfor the Mo K-edge.

Theoretical CA-EXAFS spectra were calculated at each iteration using the ab initio real-space multiple-scattering FEFF8.5L code [33,34] for the given structure model. The scattering amplitude and phase shift functions required for the simulation were calculated using the complex exchange-correlation Hedin–Lundqvist potential [35]. The calculations of the cluster potentials were carried out in the muffin-tin (MT) self-consistent field approximation using the default values of the MT radii, as implemented in the FEFF8.5L code [33].

**Figure 3:**
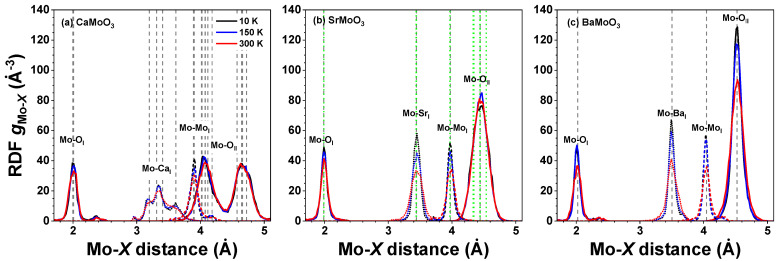
Radial distribution functions (RDFs) $g_{\mathrm{Mo}-X}\left(r\right)$ in polycrystalline (**a**) CaMoO_3_, (**b**) SrMoO_3_, and (**c**) BaMoO_3_ at 10 K, 150 K, and 300 K. Green and grey vertical dashed lines correspond to the average inter-atomic distances as obtained by XRD at 5 and 300 K, respectively. See text for details.

**Figure 4:**
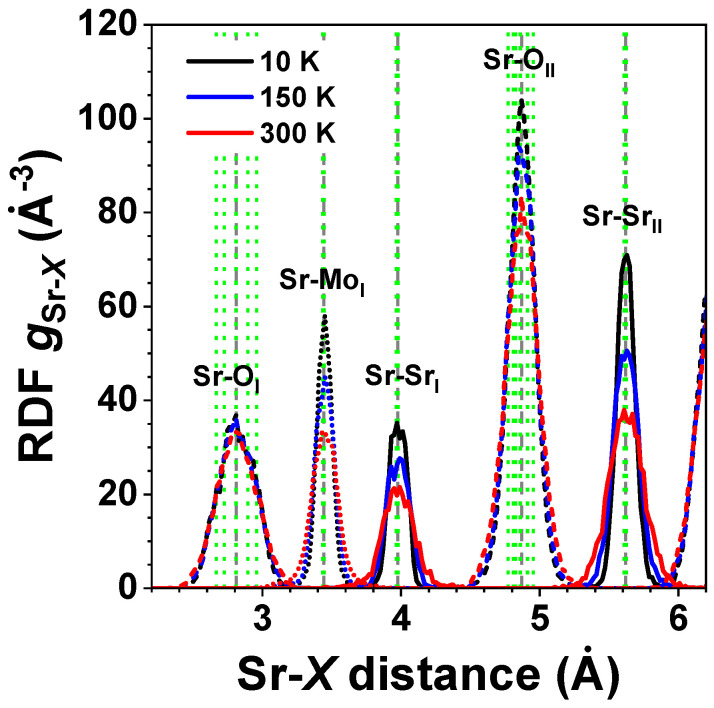
Radial distribution functions (RDFs) $g_{\mathrm{Sr}-X}\left(r\right)$ in polycrystalline SrMoO_3_ at 10 K, 150 K, and 300 K. Green and grey vertical dashed lines correspond to the average inter-atomic distances as obtained by XRD at 5 and 300 K, respectively. See text for details.

The atomic configurations obtained from the RMC/EA simulations were used (i) to calculate the partial radial distribution functions (RDFs) for metal–oxygen and metal–metal atom pairs (Figure 3 and Figure 4), (ii) to calculate the angular distribution functions (ADFs), and (iii) to perform polyhedra analysis. To improve the statistics, the distribution functions were averaged over nine simulations performed using different initial random seed numbers.

## 3. Results and Discussion

The experimental and RMC/EA-calculated EXAFS spectra at the Mo K-edge (also at the Sr K-edge for SrMoO_3_) and their Fourier transforms (FTs) for *A*MoO_3_ (*A* = Ca, Sr, Ba) perovskites at 10, 150, and 300 K are shown in Figure 2. A good agreement between the experimental and calculated EXAFS data was obtained for all samples at each temperature in the *k*- and *R*-spaces simultaneously. The wide Δk and ΔR ranges of the experimental EXAFS spectra used in the analysis allowed us to probe equally well the contributions from light oxygen and heavy metal atoms; thus, reliable structural models were obtained for different compounds and temperatures. Because a high-quality experimental Sr K-edge EXAFS spectrum can be measured in a wide energy range, SrMoO_3_ was the only compound in which the EXAFS spectra of both *A* and *B* cations were analyzed. Therefore, the obtained partial RDFs (vide infra), as seen from the *A* cation site, are more reliable for SrMoO_3_ than for CaMoO_3_ and BaMoO_3_.

### 3.1. Analysis of RDFs

Atomic coordinates in the final configurations obtained from the RMC/EA simulations were used to calculate partial RDFs for different atom pairs of interest. The temperature-dependent RDFs $g_{\mathrm{Mo}–O}\left(r\right)$, $g_{\mathrm{Mo}–A}\left(r\right)$, and $g_{\mathrm{Mo}–\mathrm{Mo}}\left(r\right)$ are shown for *A*MoO_3_ (*A* = Ca, Sr, Ba) at 10, 150, and 300 K in Figure 3, whereas the RDFs $g_{\mathrm{Sr}–O}\left(r\right)$, $g_{\mathrm{Sr}–\mathrm{Mo}}\left(r\right)$, and $g_{\mathrm{Sr}–\mathrm{Sr}}\left(r\right)$ are shown for SrMoO_3_ in Figure 4. The effect of thermal disorder on the RDF shape is clearly seen at higher temperatures; it manifests itself in the form of larger peak broadening. However, note that the thermal disorder is present even at low temperatures due to the zero-point motion of atoms [36].

**Figure 5:**
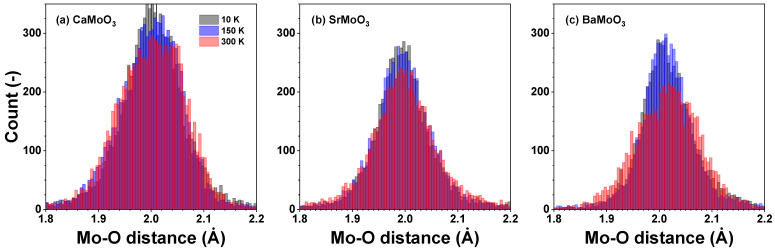
Histograms of the Mo–O distances—cf. radial distribution functions (RDFs) $g_{\mathrm{Mo}–O}\left(r\right)$—in polycrystalline (**a**) CaMoO_3_, (**b**) SrMoO_3_, and (**c**) BaMoO_3_ at 10 K, 150 K, and 300 K. See text for details.

As is evident in Figure 3 and Figure 4, the first coordination shell of Sr was much broader than that of Mo, even at 300 K. This shows that strong Mo–O bonds in [MoO_6_] octahedra have a predominantly covalent nature, whereas the Sr–O bonding has a more ionic nature. Note that the average Mo–O distances in the first coordination shell in all compounds were little affected by the type of *A* cation and temperature, cf. also histograms in Figure 5. Interestingly, the shape of the first shell RDFs $g_{{\mathrm{Sr}–O}_{I}}\left(r\right)$ remained almost the same at all temperatures in spite of the symmetry lowering below 266 K in SrMoO_3_ [11]. This fact can be explained by the interplay between the static distortions and thermal disorder; the former were larger at low temperatures in less symmetric phases but vanished at 300 K in the cubic phase, whereas the latter increased with the temperature. Thus, the changes due to the two effects compensated each other, leading to a nearly unchanged shape of the RDFs $g_{{\mathrm{Sr}–O}_{I}}\left(r\right)$.

As mentioned above, the structure of BaMoO_3_ was cubic in the studied temperature range: above 266 K, SrMoO_3_ existed in the regular perovskite structure, but below 266 K, it reduced its symmetry by tilting [MoO_6_] octahedra. This can be seen from the analysis of the RDFs $g_{{\mathrm{Mo}–O}_{\mathrm{II}}}\left(r\right)$ for the second coordination shell of Mo in SrMoO_3_ and BaMoO_3_ shown in Figure 3b and Figure 3c, respectively. According to the neutron diffraction data [11] for SrMoO_3_ at 10 K (150 K), there are 24 = 6 × 4 (24 = 2 × 4 + 2 × 8) oxygen atoms at six (four) distinct distances in the range of 4.33–4.54 Å (4.34–4.54 Å), whereas at 300 K, all 24 oxygen atoms are located at the same distance of 4.44 Å. At all temperatures, the RDF $g_{{\mathrm{Mo}–O}_{\mathrm{II}}}\left(r\right)$ peak in SrMoO_3_ is much broader than that in the cubic perovskite BaMoO_3_, and its shape is less sensitive to temperature changes due to a static distribution of Mo–O${}_{\mathrm{II}}$ distances. In cubic BaMoO_3_, all 24 oxygen atoms are located at the same distance of 4.52 Å [9,10].

### 3.2. Polyhedral Analysis

The coordinates of atoms in the RMC simulation box were used to evaluate and statistically analyze the geometry of the [MoO_6_] coordination polyhedra in terms of the Mo–O distances, the inter-octahedral Mo–O–Mo and intra-octahedral O–Mo–O angles, and several distortion parameters [37,38].

It is recognized that single parameter approximations used to describe polyhedra and their distortions are useful, but commonly used functions based on inter-atomic distances are not sensitive to angular distortions, whereas angular variance functions ignore distance variations [39].

Following the approach developed in [38], we used the ζ, Σ, Θ, and Δ parameters to characterize the stretching, angular, torsional, and tilting distortion of [MoO_6_] octahedra, respectively. These parameters are defined as follows:

(1) $$\zeta =\sum_{i=1}^{6}|d_{i}-\langle d\rangle |,$$

(2) $$\Sigma =\sum_{i=1}^{12}|\varphi_{i}-90^{{}^{\circ}}|,$$

(3) $$\Theta =\sum_{i=1}^{24}|\theta_{i}-60^{{}^{\circ}}|,$$

(4) $$\Delta =\frac{1}{6}\sum_{i=1}^{6}{\left(\frac{d_{i}-\langle d\rangle }{\langle d\rangle }\right)}^{2},$$

where $d_{i}$ and 〈d〉 are the unique Mo–O inter-atomic distances and the mean inter-atomic position within an octahedron, respectively, $\varphi_{i}$ are the O–Mo–O angles within an octahedron, and $\theta_{i}$ is the unique torsional angle viewed along the pseudo-threefold axis in an octahedron. Note that for a regular octahedron, all these parameters are equal to zero.

In perovskites, the geometries around molybdenum atoms can significantly deviate from regular octahedra depending on the nature of the chemical bonds (static distortion) and thermal motion (dynamic disorder). The general shape of the histograms of Mo–O distances is temperature dependent: a pronounced skewness of the ${\mathrm{Mo}–O}_{I}$ distance distribution in CaMoO_3_ (Figure 3a and Figure 5a) demonstrates the perturbation of the local octahedral coordination of Mo^4+^ ions, which increases with increasing temperatures. More symmetric ${\mathrm{Mo}–O}_{I}$ distance distributions in SrMoO_3_ and BaMoO_3_—see Figure 3b,c and Figure 5b,c—demonstrate a more regular local octahedral coordination of Mo^4+^ ions. The local geometry of molybdenum sites is more sensitive to temperature changes in BaMoO_3_, as also confirmed by the changes in the distortion parameters (see below).

**Figure 6:**
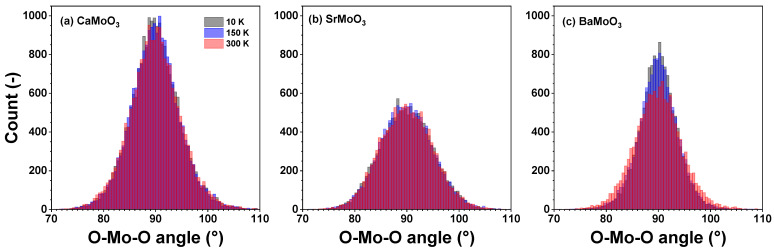
Histograms of intra-octahedral O–Mo–O angles—angular distribution functions (ADFs)—in polycrystalline (**a**) CaMoO_3_, (**b**) SrMoO_3_, and (**c**) BaMoO_3_ at 10 K, 150 K, and 300 K. See text for details.

**Figure 7:**
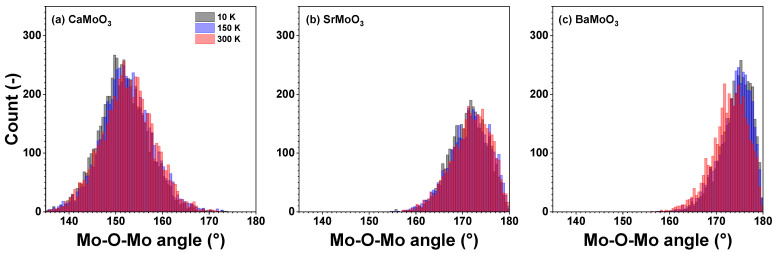
Histograms of inter-octahedral Mo–O–Mo angles—angular distribution functions (ADFs)—in polycrystalline (**a**) CaMoO_3_, (**b**) SrMoO_3_, and (**c**) BaMoO_3_ at 10 K, 150 K, and 300 K. See text for details.

The distributions of the intra-octahedral O–Mo–O and inter-octahedral Mo–O–Mo angles in CaMoO_3_, SrMoO_3_, and BaMoO_3_ are shown in Figure 6 and Figure 7, respectively. The average intra-octahedral O–Mo–O angle is equal to 90${}^{{}^{\circ}}$ for all three compounds and depends weakly on the temperature.

In cubic perovskites, the value of the average inter-octahedral angle is always smaller when calculated from the mean distances (from EXAFS data) than from the average positions of the atoms (from diffraction data) due to the tilting motion of octahedra [25,30,40,41]. Indeed, in the cubic BaMoO_3_, the average inter-octahedral Mo–O–Mo angle, as determined by RMC-EXAFS, is close to 175${}^{{}^{\circ}}$, and the mode of the angle distribution slightly decreases with increasing temperatures due to the raising amplitude of the tilting motion of [MoO_6_] octahedra. The reduction of the Mo–O–Mo angle below 180degrees should affect the conduction band, which appears as a hybridization of the p(O) and the e${}_{g}$ levels of Mo.

In SrMoO_3_, the average inter-octahedral Mo–O–Mo angle, as determined by RMC-EXAFS, is close to 171${}^{{}^{\circ}}$, and the mode of the angle distribution is not sensitive to the temperature changes, whereas the angle value determined from the neutron diffraction studies is equal to 171–173${}^{{}^{\circ}}$ in the low-temperature orthorhombic phase at *T* < 125 K but to 180${}^{{}^{\circ}}$ in the tetragonal (125 K ≤T≤266 K) and cubic (*T* > 266 K) phases [11].

In CaMoO_3_, the inter-octahedral Mo–O–Mo angle, as determined from the diffraction data, is about 153${}^{{}^{\circ}}$ due to the tilted [MoO_6_] octahedra (leading to the orthorhombic symmetry, Ref. [12]). The average value of the angle, as determined by RMC-EXAFS, is close to 150${}^{{}^{\circ}}$ and increases slightly with increasing temperatures.

The changes in the average inter-octahedral angle in the series CaMoO_3_–SrMoO_3_–BaMoO_3_ can be explained by the structural changes induced by the different sizes of *A*-ions: smaller *A*-ions cannot fill the space between the octahedra and oxygen ions tend to move toward the cube centre; this leads to reducing the *A*–O distance (Mo–O also changes at the same time) and the Mo–O–Mo angle below 180${}^{{}^{\circ}}$. This correlates with the resistivity data in these compounds: as the inter-octahedral angle becomes less than 180${}^{{}^{\circ}}$, the hopping probability for carriers to move from Mo to Mo sites decreases [5,42] due to a reduction in the overlap of the d(Mo) and p(O) wave functions, leading to an increase in the charge localization and a reduction in the carrier mobility.

**Figure 8:**
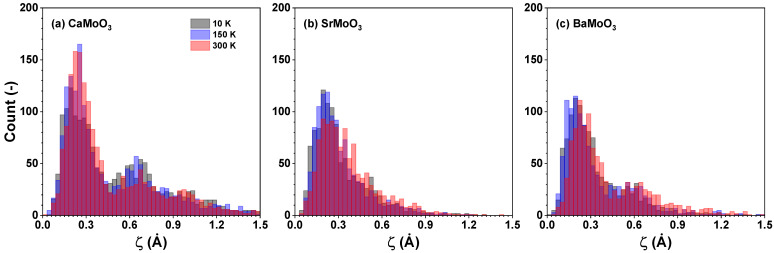
Histograms of calculated ζ values (Equation (1)) describing the stretching distortion of [MoO_6_] octahedra in polycrystalline (**a**) CaMoO_3_, (**b**) SrMoO_3_, and (**c**) BaMoO_3_ at 10 K, 150 K, and 300 K. See text for details.

**Figure 9:**
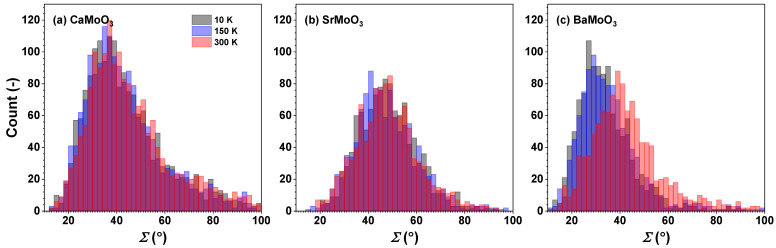
Histograms of calculated Σ values (Equation (2)) describing the angular distortion of [MoO_6_] octahedra in polycrystalline (**a**) CaMoO_3_, (**b**) SrMoO_3_, and (**c**) BaMoO_3_ at 10 K, 150 K, and 300 K. See text for details.

**Figure 10:**
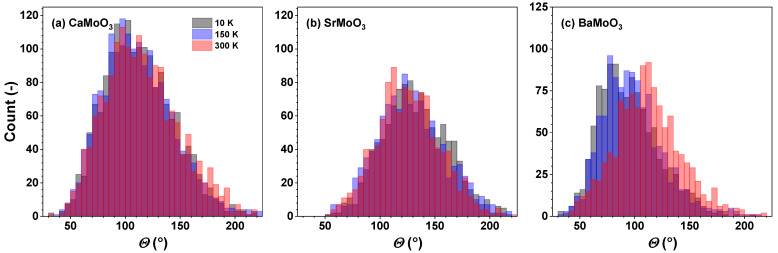
Histograms of calculated Θ values (Equation (3)) describing the torsional distortion of [MoO_6_] octahedra in polycrystalline (**a**) CaMoO_3_, (**b**) SrMoO_3_, and (**c**) BaMoO_3_ at 10 K, 150 K, and 300 K. See text for details.

[MoO_6_] octahedra can also be subject to stretching distortions. Nevertheless, the degree of (temperature-induced) stretching distortion ζ of [MoO_6_] octahedra is small and comparable in all three compounds (Figure 8). It shows almost no temperature dependence in the studied temperature range. In SrMoO_3_, [MoO_6_] octahedra are characterized by large angular distortions Σ (Figure 9), which are not temperature sensitive. At the same time, in BaMoO_3_, the angular Σ and torsional Θ distortions of [MoO_6_] octahedra exhibit a pronounced temperature dependence (Figure 9 and Figure 10).

**Figure 11:**
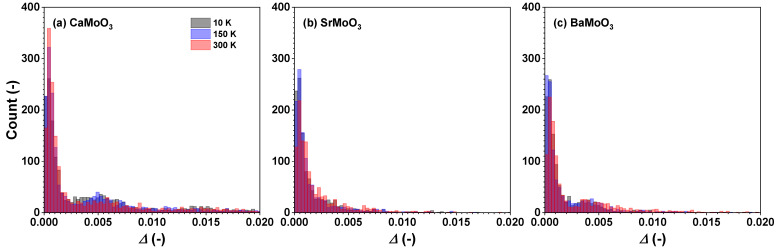
Histograms of calculated Δ values (Equation (4)) describing the tilting distortion of [MoO_6_] octahedra in polycrystalline (**a**) CaMoO_3_, (**b**) SrMoO_3_, and (**c**) BaMoO_3_ at 10 K, 150 K, and 300 K. See text for details.

In all three compounds, the tilting distortions of [MoO_6_] octahedra are small and comparable in magnitude (Figure 11) and show almost no temperature dependence in the studied temperature range.

## 4. Conclusions

The reverse Monte Carlo method with the evolutionary algorithm was used to analyze temperature-dependent (10–300 K) extended X-ray absorption fine structure spectra of *A*MoO_3_ (*A* = Ca, Sr, Ba) perovskites with the goal of revealing the influence of thermal disorder and static distortions on their local atomic structure up to 5–6 Å around the absorbing centre. The coordinates of atoms obtained during the reverse Monte Carlo simulation were employed to evaluate and statistically analyze the geometry of [MoO_6_] octahedra in terms of the Mo–O distances, the inter-octahedral Mo–O–Mo and intra-octahedral O–Mo–O angles, and a set of distortion parameters, which characterize stretching the (ζ), angular (Σ), torsional (Θ), and tilting (Δ) distortions of [MoO_6_] octahedra, as defined by Equations (1)–(4).

[MoO_6_] octahedra behave as fairly rigid structural units in the three perovskites studied, which, however, show different responses when cooled below room temperature. In BaMoO_3_, the thermal disorder appears in all types of [MoO_6_] distortions (the stretching, angular, torsional, and tilting) (Figure 8, Figure 9, Figure 10 and Figure 11); this also affects the distance distributions (Figure 3 and Figure 5) and the intra- and inter-octahedral angular distribution functions (Figure 6 and Figure 7). In SrMoO_3_, by lowering the experimental temperature, an increase in the stretching distortions of [MoO_6_] octahedra (Figure 3, Figure 5 and Figure 8) was observed; this can be explained by two phase transitions during cooling (from the cubic phase to the tetragonal phase below 266 K, and, next, to the low-temperature orthorhombic phase below 125 K). Finally, [MoO_6_] octahedra in CaMoO_3_ demonstrate the weakest sensitivity to temperature variations because CaMoO_3_ remains in the orthorhombic phase with a cooperative tilting of [MoO_6_] octahedra.

In conclusion, we demonstrated the ability of an accurate analysis of extended X-ray absorption fine structure data based on the reverse Monte Carlo method to provide detailed information on the local lattice dynamics in perovskite compounds. In particular, the evolution of the distortion of the coordination sphere of [MoO_6_] octahedra in functional oxide materials has been speculated for a while but, to the best of our knowledge, has not yet been proved. This example clearly demonstrates the richness of the perspectives offered by polyhedral analysis in conjunction with large datasets, as often found in RMC analysis.

## Data Availability

The data presented in this study are available on request from the corresponding author.

## Abbreviations

The following abbreviations are used in this manuscript:

ADF	angular distribution function
CA	configuration-averaged
EA	evolutionary algorithm
EXAFS	extended X-ray absorption fine structure
FT	Fourier transform
MT	muffin-tin
RDF	radial distribution function
RMC	reverse Monte Carlo
XAS	X-ray absorption spectroscopy
